# A review of research progress in the design history of everyday life

**DOI:** 10.1016/j.heliyon.2024.e40865

**Published:** 2024-12-11

**Authors:** Hongying Jia

**Affiliations:** Tongji University, College of Design and Innovation, Shanghai, China

**Keywords:** Design history, Everyday life, Bibliometrics, Knowledge graph, Research progress

## Abstract

The study of everyday life has garnered significant research attention in various disciplines. However, in the field of design history, the exploration of everyday life remains in its early stages. There is a need for further organization and analysis, as there is currently no comprehensive exposition on the overall research progress in this field. To address this gap, this paper employs bibliometric and knowledge graph methods to summarize the research progress in the design history of everyday life. By analyzing high-frequency keywords, thematic trends, citation networks, co-occurrence knowledge graphs, and burst keywords, we provide an objective evaluation of the development of the research perspective on everyday life in design history. The findings reveal an overall increasing trend in research efforts in the design history of everyday life, with a focus on Europe and America. The research in this field has evolved from philosophical explorations to disciplines such as history and anthropology, with interconnections between different directions. Prior to 2000, the emphasis was on the philosophical essence and fundamental theories of everyday life. From 2001 to 2010, there was a surge in psychological research and discussions on female topics within the design history of everyday life. In recent years, there has been a further expansion in the proportion of research on everyday life in the design history field, with more diverse dimensions of study. However, it is important to note that the research in the design history of everyday life is currently in a stage of conceptual understanding, with limited theoretical achievements. This paper not only provides a comprehensive overview of the current research progress but also presents an outlook on the research prospects in the design history of everyday life. It serves as a valuable resource for scholars and practitioners interested in advancing the understanding of the design history of everyday life.

## 1 Introduction

The study of design history has traditionally focused on prominent figures and their major works, but the nature of the design discipline determines the difficulty in obtaining historical materials. Therefore, a shift in research perspective is deemed more important. Everyday life is the material structure in which cultural and economic expressions occur, and it is crucial for the formation of the self and society. Under the influence of philosophical trends, everyday life has become an important research object and method in various fields such as history and folklore, presenting a vivid and three-dimensional historical state through insights into micro-level life and conveying relatively stable design genes. Therefore, everyday life should be an effective reference perspective for exploring, constructing, and reconstructing design history, bearing positive significance for discovering the value and connotation of design history, as well as sorting, summarizing, and developing design history.

In the early 20th century, the concept of "everyday life" first appeared in the interpretation of the "lifeworld" by Austrian writer and philosopher Husserl, who stated, "It is the existence of experience, which is what we call the everyday life world. We take it for granted in our daily lives, we are very familiar with it, and we never question it [1]." In the 1940s, Henri Lefebvre further developed the concept, introducing the idea of critical everyday life. He argued that everyday life is often perceived as trivial and insignificant, leading to a separation between intellectual philosophy and the sensory world of everyday life. The critique of everyday life thus aims to challenge this alienation [2]. As can be seen, the universality and accessibility of everyday life offer philosophers the possibility to unearth new insights and perspectives from overlooked human experiences, revealing the potential structures and patterns in the seemingly ordinary life. On the other hand, philosophers use everyday life as a tool to critique society and culture, aiming to reveal how power structures, ideologies, and social norms shape our everyday lives.The perspective of "everyday life" has also made significant contributions to various fields of study. In the field of history, it has evolved from the Annales School [3,4] to Microhistory [5,6] and the New Cultural History [7,8]. Scholars have utilized this perspective to delve into the lives of ordinary people, uncovering important cultural information and enriching our understanding of the past. In folklore studies, figures like Bausinger have advocated for an exploration of the "folk culture of the technological world," aiming to analyze and convey cultural significance through marginalized figures or events [9]. Additionally, in sociology, Michel Foucault has explored the everyday life world of modern society, offering contrasting perspectives to phenomenological subjectivism and conservatism [10,11].

As the study of "everyday life" continues to gain traction and influence, it has intersected with design history in increasingly meaningful ways. This convergence has given rise to the emerging field of the design history of everyday life, exploring topics such as female's design, micro-design, the aestheticization of everyday life, and the redesign of everyday objects. In the realm of industrial design history, John Heskett highlights the significance of design as a fundamental form of human expression that encompasses functionality and aesthetics [12,13]. Even seemingly simple objects can be designed to reflect specific cultural contexts. This prompts design historians to pay closer attention to the ordinary objects that surround us, recognizing their potential for cultural analysis. The selection of historical materials poses a challenge for design historians. Jonathan M. Woodham, in his book *Twentieth-Century Design*, openly acknowledges the difficulties in finding sufficient evidence for research when faced with crucial archival and museum materials [14]. To write a more comprehensive design history, it becomes crucial to give fair consideration to various patterns of consumption and everyday life, shifting away from the traditional emphasis on cultural elites and chronological frameworks. This shift in focus has popularized the notion that the most famous designs of the 20th century are not confined to museums but exist within the marketplace, reflecting the impact and relevance of everyday life in design history.

It's worth noting that among the various research fields of design history of everyday life, design history of everyday life shares a more intimate connection with microhistory. Both start from grassroots, individual perspective, focusing on those often overlooked, seemingly insignificant matters. design history of everyday life, by paying attention to everyday life items related to design, reveals how these items shape our living environment, habits, and in turn, influence the development of design. However, we must also recognize that this excessive focus on the 'minor' may overlook large-scale social, political, and economic processes, leading to a one-sided understanding of history. Therefore, we need to maintain critical thinking, avoid trivial and fragmented research, and strive to find a balance between the micro and macro levels to more comprehensively understand the complexity of design history of everyday life and microhistory.

The interdisciplinary nature of design and the emerging research perspective of everyday life pose challenges to the measurement and evaluation of design history. Design history of everyday life mainly relies on historical methods and focuses on specific design cases, which limits a comprehensive review and reflection on the overall progress of this field.

Bibliometric methods, on the other hand, can serve as a research tool for the academic study of design history, providing a comprehensive review and reflection. It can not only capture research trends, discover new research themes, and assess research impact, but also, in terms of interdisciplinary studies, bibliometrics can reveal the integration of design history with other disciplines through the analysis of literature citation networks. This facilitates a clearer understanding of the relationships between disciplines and promotes the development of interdisciplinary research in design history. It is evident that design history of everyday life resides at the intersection of design history and other disciplines.

Therefore, this paper employs bibliometric research tools to conduct a comprehensive review of publications and examines the development of design history of everyday life. It uses software like CiteSpace, VOSviewer, and HisCite to analyze publications related to the Web of Science database from 1990 to 2022. By using these analytical tools, this paper aims to clearly understand the current research progress in this field, identify the latest research trends, find research gaps, and provide visual data references for scholars interested in this research area.

## 2 Data collection and metrology methods

### 2.1 Data collection

Web of Science is a large-scale, comprehensive, multidisciplinary, core journal citation index database, including three major citation databases (SCI, SSCI, and A&HCI) and two factual chemical information databases (CCR and IC). It is often used for bibliometric analysis and is one of the most comprehensive and widely used high-quality databases in bibliometric analysis. It is now widely accepted by researchers worldwide and has become a standard tool for searching and evaluating different types of publications. Due to its academic rigor, data consistency, and stability, it is widely used as a dataset for large-scale data-intensive research in various fields [[15], [16], [17]]. Therefore, we chose Web of Science to search and collect all types of literature related to this study. In this study, the time interval is set from January 1990 to December 2022 to capture relevant publications on the design history of everyday life. A thematic search is performed on the Web of Science platform, utilizing the search query TS= ("daily life" or "everyday life") AND TS= (design∗ or plan∗ or devise∗) AND TS=(history∗). This query is designed to retrieve publications that specifically addresses the intersection of daily life and design history. To ensure the accuracy and relevance of the search results, additional criteria are applied to exclude unrelated fields such as public administration, materials science, and nutrition. This refinement aims to focus the analysis on publications directly related to the design history of everyday life. As a result of this comprehensive search strategy, a total of 1785 publications records are retrieved, and these publications were saved as text ﬁles containing “Full Record and Cited References”, providing a robust dataset for further analysis and examination.

### 2.2 Metrology methods

This study employs a multi-faceted approach to analyze the collected publications in the field of the design history of everyday life. Firstly, the collected publications is organized and analyzed in terms of quantity, time, country, and authors. This analysis provides specific data on the publication output, countries with the highest research activity, prolific authors, influential journals, and leading research organizations in the field. Visual representations such as charts and graphs are created to offer a clear overview of the overall research status in the design history of everyday life.

Secondly, comprehensive bibliometric analyses are conducted using specialized software such as CiteSpace, VOSviewer, and HisCite. These tools enable the exploration of high-frequency keywords, thematic trends, research evolution, and the identification of emergent keywords in the field. By employing statistical analyses and generating knowledge maps, this study provides detailed insights into the dynamics and development of research in the design history of everyday life.

Lastly, to complement the bibliometric research methods, this study incorporates a comprehensive analysis of the Journal of Design History. By examining the content of this influential journal in the field, the study ensures a more comprehensive and nuanced understanding of the research landscape in the design history of everyday life. This supplementary analysis further enriches the findings and contributes to a more holistic examination of the field.

## 3 Results and discussion

### 3.1 Overall overview (number of publications, research countries, authors, journals and organizations)

**Fig. 1:**
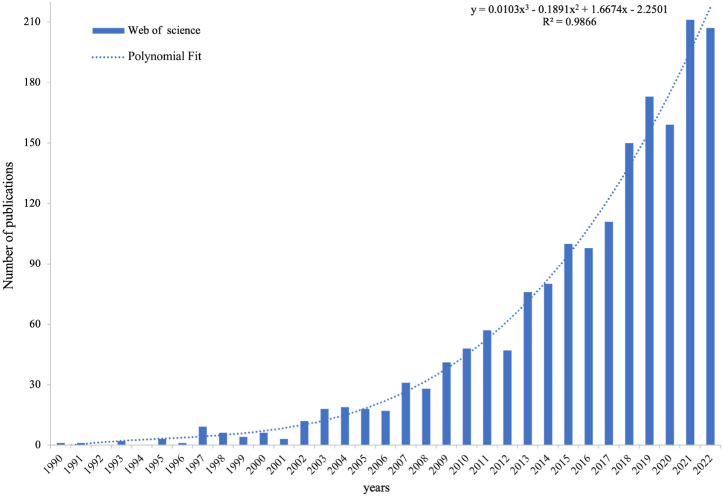
Annual trend of the design history of everyday life research publications from 1990 to 2022.

The preliminary analysis and statistical calculations of the 1785 publications records from the Web of Science database reveal trends of the number of publications. Fig. 1 visually represents the results. From the polynomial fit of the data, it is evident that the number of publications records in this field has been increasing over time. This upward trend indicates a growing interest and recognition of the importance of studying the design history of everyday life. Furthermore, the research trend depicted in Fig. 1 shows a significant rise in global studies on the design history of everyday life after 2010. This indicates that this research topic has gained prominence and become a focal point for scholars and experts worldwide. The increasing number of publications records suggests a growing recognition of the significance of everyday life design in shaping societies and cultures.

In recent decades, research in design history of everyday life has shown a growing trend, which may be driven by several factors. Firstly, a deeper understanding of design elements in daily life has led to a demand for improving our everyday experiences, prompting more researchers to delve into design history of everyday life studies. Secondly, with the diversification of historical research methods, microhistory or minimalist history methodologies are garnering more attention. As an application field of microhistory research, design history of everyday life naturally benefits from this trend. Lastly, as society and culture progress, our focus and understanding of everyday life continually deepen, drawing more researchers' attention to design history of everyday life studies.

**Fig. 2:**
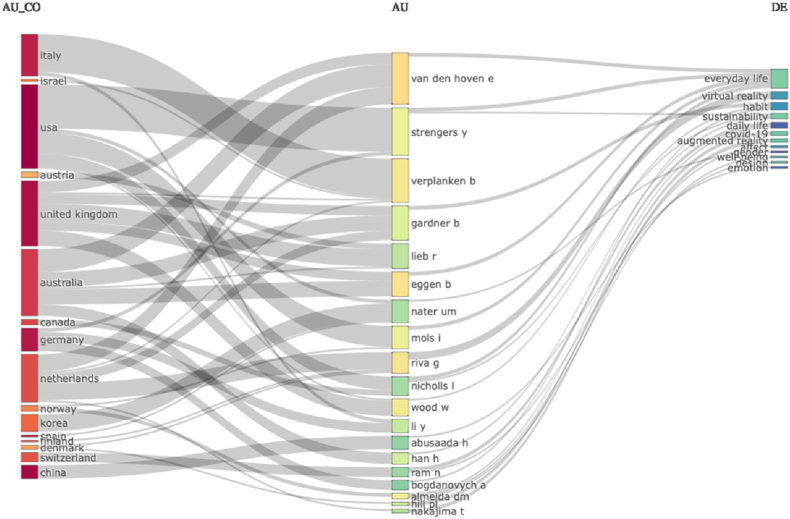
Three-field map of major research countries, authors, keywords in the design history of everyday life research publications.

According to the statistical analysis of the country distribution of the 1785 publications records in Web of Science, it is found that the United States has the highest number of publications with 299 papers, accounting for 16.75 % of the total. Following that, the United Kingdom has 199 papers (11.14 %), and Germany has 149 papers (8.35 %). Italy, Australia, and China also have more than 80 publications each. Overall, it can be observed that research on the design history of everyday life is primarily concentrated in developed countries, while developing countries are still in the preliminary stages. Additionally, notable researchers in the field of the design history of everyday life include Wood W, Verplanken B, Eggen B, Riva G, Van Den Hoven E, Almeida DM, Mols I, Strengers Y, Carbon CC, Scott K, and others (Fig. 2).

From the perspective of publication journals, research on the design history of everyday life is dispersed among various journals (Table 1). While there is no specific journal that dominates the field, there are several notable journals where a significant number of publications can be found. The top-ranking journal in terms of the number of publications is Sustainability, with 21 publications records. This journal demonstrates a focus on the intersection of design history and sustainable practices. Following closely is the International Journal of Art & Design Education, with 4 publications, indicating a strong emphasis on the educational aspects of the design history of everyday life. Journal of Environmental Psychology also emerges as a prominent journal in this field, with 3 publications. This journal explores the psychological aspects of design and its impact on everyday life experiences. Design and Culture, despite appearing in the top five, only has 2 publications, suggesting that research in the design history of everyday life has not received sufficient attention in design history journals.

**Table 1:** Top 5 journals in the design history of everyday life research publications.

No.	Journal title	Quantity	Proportion/%
1	Sustainability	21	3.46 %
2	International Journal of Art & Design Education	4	0.66 %
3	Journal of Environmental Psychology	3	0.49 %
4	Design and Culture	2	0.33 %
5	Urban Studies	2	0.33 %

^1^ The leading international journals in the statistical analysis may not be closely related to design or design history itself. The data in this table is the result of secondary screening of core and important journals.

The research achievements in the design history of everyday life are relatively dispersed among different journals. This indicates the interdisciplinary nature of the field and the need for researchers to engage with various disciplines and publication outlets. It also suggests that there is room for further exploration and contribution in design history journals to enhance the understanding and appreciation of the design history of everyday life.

In terms of research organizations, the University of London has the highest number of research outputs in the field of everyday life design, with a total of 39 articles, accounting for 2.18 % of the total. Other prominent organizations in this field include the University of California, University College London, the University of Manchester, and the University of Zurich (Table 2).

**Table 2:** Top 5 organizations in the design history of everyday life research publications.

No.	Organizations	Quantity	Proportion/%
1	University of London	39	2.18 %
2	University of California	23	1.29 %
3	University College London	20	1.12 %
4	University of Manchester	15	0.84 %
5	University of Zurich	15	0.84 %

Interestingly, the analysis indicates that international research on the design history of everyday life is primarily concentrated in comprehensive universities rather than art schools or specialized organizations. It emphasizes the importance of comprehensive universities in fostering research and contributing to the understanding of the design history of everyday life.

### 3.2 High-frequency keywords

**Fig. 3:**
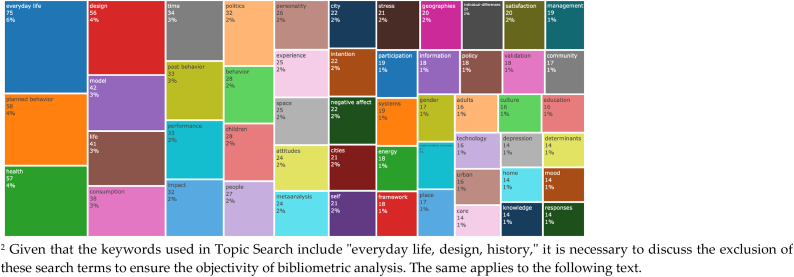
High-frequency keywords in the design history of everyday life research publications.

High-frequency keywords are critical terms that demonstrate a noticeably increased frequency or usage. Conducting statistical analysis of these keywords allows for a deeper exploration of the forefront dynamics in the field. After filtering out keywords with frequencies less than 14, a total of 53 high-frequency keywords were identified (Fig. 3).

Through an analysis of high-frequency keywords, it is observed that "planned behavior" and "health" are among the most frequently mentioned terms. Further exploration of relevant publications reveals that this is due to the focus of everyday life design in the medical field, such as monitoring the daily activities of elderly individuals and designing assistive devices [[18], [19], [20]]. Moreover, words like "model," "consumption," and "space" also have relatively high frequencies, indicating the significance of everyday life design in urban development, spatial design, and the construction of modernity [21,22]. It is evident that research on everyday life design remains primarily focused on specific case designs, aiming to improve various aspects of daily life through design interventions, without yet encompassing the macro-level perspective of "design history".

**Fig. 4:**
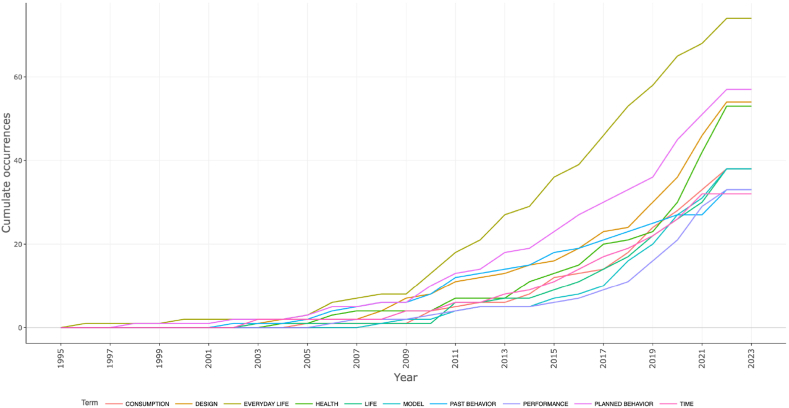
High-frequency keyword time trends in the design history of everyday life research publications.

However, it is important to note that while high-frequency keywords provide insights into popular terms, they may not capture the changing trends and dynamics of these keywords over time. To address this limitation, further research is conducted to examine the temporal trends of the top 10 high-frequency keywords, as depicted in Fig. 4.

By considering the temporal trends of high-frequency keywords, it is evident that "planned behavior" has shown a consistent upward trajectory since 2009. On the other hand, the frequency of "health" has fluctuated with an overall increasing trend since 2011, which further accelerated from 2019 onwards. Comparing Fig. 3, Fig. 4, it is observed that while both "performance" and "past behavior" have occurred 33 times, "performance" surpassed "past behavior" around 2010 and has remained relatively stable until 2022. From this, it can be inferred that in the design history of everyday life, there is a persistent interest in the research related to the detection of human daily behaviors and health, as well as the study of performance aspects of everyday objects.

### 3.3 Thematic trends

**Fig. 5:**
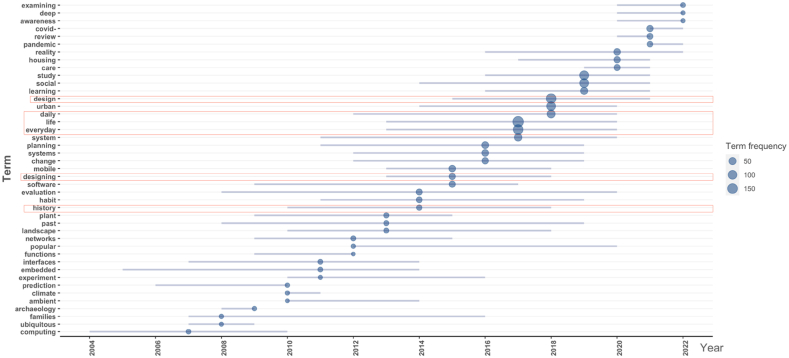
Thematic trends in the design history of everyday life research publications.

Thematic trends offer a forecast of future hot topics and provide an overview of the overall grasp and direction of publications themes. Given that the title of a paper succinctly summarizes its main content, a statistical analysis of the themes of the 1785 documents from WOS has been conducted, and the results are presented in Fig. 5.

In the realm of thematic trends, the emergence of the term "pandemic" in the field of the design history of everyday life can be attributed to the global context of the COVID-19 outbreak that began in 2019 [23,24]. Additionally, the high-frequency themes of "study" and "social" reached their peak in 2019, while "urban" and "learning" were most prominent in 2018. By combining the analysis of high-frequency keywords and relevant publications, it is evident that design history education [[25], [26], [27], [28]] and urban design [29,30] were popular research topics in the field of the design history of everyday life during 2017–2020. Based on Fig. 5, it can be speculated that a retrospective exploration of the design history of everyday life and the study of ordinary people's living spaces may become future focal points in the research on the design history of everyday life.

### 3.4 Citation network

**Fig. 6:**
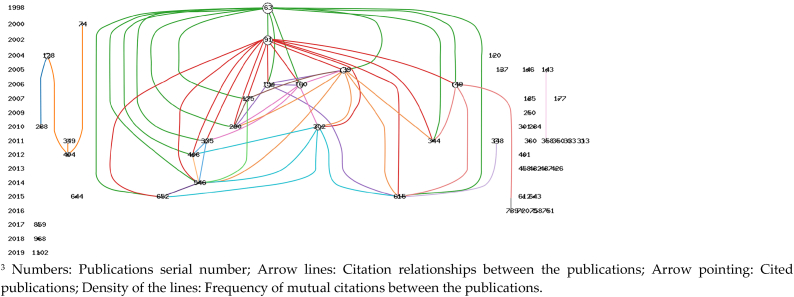
Citation network in the design history of everyday life research publications

The citation timeline chart created for the international publications on the design history of everyday life using HistCite software reveals interconnected research themes with strong cross-referencing relationships (see Fig. 6).

1990–2000: Before 1998, there are no citation nodes, indicating the absence of highly cited papers in the initial 8 years. The earliest node in the past 30 years is publications 63, published in 1996, and is the most highly cited publications with 44 citations. Authored by Ouellette JA and Wood W, titled "Habit and intention in everyday life: The multiple processes by which past behavior predicts future behavior," this paper offers a psychological perspective and integrates the theory of past behavior guidance. It suggests that the frequency of past behavior reflects the strength of habits and constructs contemporary social psychology human behavior models through the explanation of everyday behavior—habit [31]0.1998–2000: During this period, one citation node appears, publications 74, which, while cited only twice among the 1785 papers, has been cited 317 times in the entire WOS database, indicating its influential status. Authored by Tennen H, this paper, also based on the psychological research direction, addresses internal issues of personnel in clinical practice. Tennen aims to use interpersonal relationship design to solve clinical problems, emphasizing the importance of everyday behavior to participants and researchers [32].In summary, this time period represents the embryonic stage of the design history of everyday life. The exploration of everyday life is limited to individual case practices within the field of psychology. The publications primarily focus on designing everyday life rather than the design of everyday life, and there is a lack of research on historical materials and theories.

During the period from 2001 to 2010, a surge in highly cited publications is observed. Among them, the most significant LCS is the 2002 publication by Wood W, Quinn JM, and Kashy DA in the "Journal of Personality and Social Psychology" titled "Habits in everyday life: Thought, emotion, and action." Wood W continues the exploration of the importance of everyday behavior in fostering habits, employing a diary system data collection method to explore strategies for using everyday behavior to reduce stress, increase efficiency, while maintaining personal emotional engagement and intentional thought [33]. Additionally, authors Verplanken B of citation node publications 154 and 160, Almeida DM of publications 137, and Gollwitzer PM of publications 149 similarly investigate everyday behavior from a psychological perspective [[34], [35], [36], [37]]. Llewellyn M of citation node publications 120 adopts a new perspective, analyzing modern architecture from the narratives of the easily overlooked first residents of the unremarkable residential area of Kensal House in 1930s Britain. This approach draws attention to many often marginalized voices in the historical narrative of modern architecture [38]. Furthermore, Fenster T of publications 146 discusses "The right to the gendered city: Different formations of belonging in everyday life" from a gender and feminist perspective, deliberating on new forms of citizenship in the globalized city. This work critically reflects on women's daily experiences and their sense of comfort and belonging in the cities they inhabit, linking these with women's everyday lives, urban planning, and governance [39]. In summary, during this period, in addition to the psychological perspective, the research perspective on everyday life is broadened. Whether from the history of architecture or other fields such as urban governance, 'overlooked' and 'marginalized' historical materials began to receive attention, bringing the study of the design history of everyday life closer to the research itself.

During the period of 2011–2020, the author with the most cited publications nodes is Strengers Y, with 3 papers (313, 333, and 1102) collectively cited 14 times, all based on anthropological research. Among them, publications 313, from the perspective of computer science, investigates household energy and water consumption in daily activities such as showering, laundry, and refrigeration. It reveals that activities are influenced by dynamic social, cultural, technological, and institutional factors and proposes an alternative design paradigm based on the reality of everyday life—an ecological feedback system [40]. Publications 333, from the angle of public administration, emphasizes the divergence between the production of energy and water and seemingly independent consumption domains, overlooking the dynamic changes in the everyday practices of resource consumption. By shifting the focus from shared resource management to shared management of everyday practices, it presents an alternative resource management paradigm, integrating the concepts of shared management and social practice theory [41]. Publications 1102, from an ecological perspective, prioritizes and limits potential futures, proposing the "social practice imagination" to develop alternative future scenarios based on changing everyday life and conducting experiments using the example scenario of "pets staying at home" [42]. During this period, Hargreaves T, focusing on law, reveals profound challenges encountered in attempting to challenge and change social theoretical practices, namely the organizational structure of everyday life, providing a more comprehensive and grounded perspective for the practice theory to the process of behavior change [43]. At the same time, design studies emerge with a fresh perspective, with Scott K's publication in "Design Studies" titled "Designing change by living change." Against the backdrop of sustainable development, it focuses on the role of technology and design work in mitigating or exacerbating consumption-related impacts, lacking the necessary systematic perspective to appropriately address the social nature of consumption. Using bathing as a case study, it explores the collaborative process of discourse analysis and experiments in everyday life [44].

It is evident that the research perspective of everyday life has undergone a developmental process, extending from psychology to architecture, anthropology, law, and then to design studies. This trend signifies the increasing influence of everyday life across diverse disciplines. However, it has not yet fully emerged in the history of design.

### 3.5 Co-occurrence knowledge graph

**Fig. 7:**
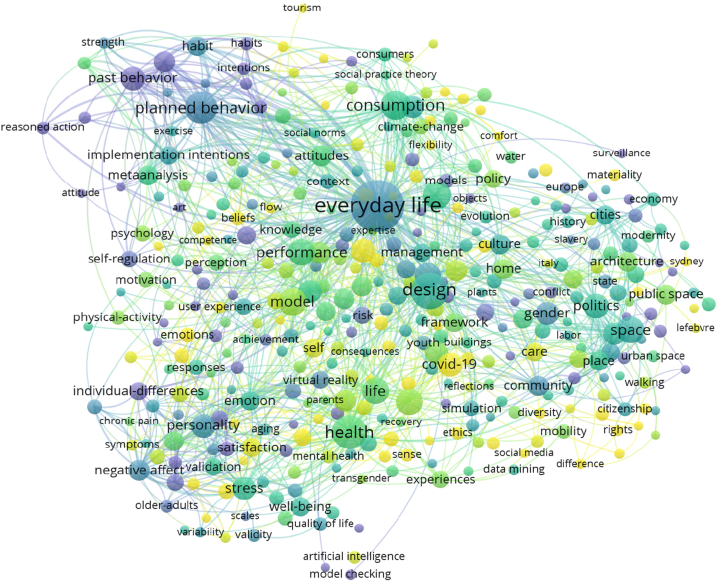
Co-occurrence Knowledge Graph in the design history of everyday life research publications.

Through the analysis of high-frequency keywords and the trend of topics, one can generally grasp the hot topics in the progress of the design history of everyday life research. However, the inherent connections between research hotspots are difficult to demonstrate. Therefore, by using VOSviewer for co-occurrence and cluster analysis, 378 keywords with frequencies higher than 5 are extracted to create a co-occurrence relationship network map as shown in Fig. 7. It is observed that.

1) "Everyday life" is at the center and is associated with other keywords to varying degrees, with the highest centrality.
2) In addition to "everyday life," "design," "health," and "consumption" also exhibit relatively high centrality. The impact of everyday life design on health care and the construction of social rights has always been an international research focus. Furthermore, it has sparked related considerations about design itself, such as John Heskett's proposition of "What is design," advocating for design to shape the quality of life. Guided by this concept, there is increased attention to the design of ordinary objects, indirectly transforming the perspective of design and enhancing awareness of everyday life [12].
3) Although keywords such as "history," "experience," and "individual difference" appear at the periphery of the co-occurrence analysis graph, the transformation of design history due to the shift in the perspective of everyday life research has taken place. This transformation will fundamentally impact the experience of everyday life design and the individual characteristics of ordinary people. Therefore, the entry of everyday life into design history and the expansion of design history from the perspective of everyday life may become hot topics for future research.

Using VOSviewer, the 376 keywords were divided into 7 clusters, with the first three clusters containing 105, 74, and 66 keywords, respectively.

Cluster 1 includes keywords such as "agriculture," "anthropology," "archaeology," "construction," and "contact," primarily discussing the relationship between everyday life and anthropology, architecture, and archaeology. For instance, anthropological scholar Burkitt I proposed that the lived experience of everyday life is multidimensional, comprising various social practice domains, each expressed, compiled, and regulated to varying degrees in different ways (official or unofficial). Therefore, everyday life is a mixture of various forms of production and connection, each uniquely combining time and space [45].

Cluster 2 includes keywords such as "design," "individual differences," "future," and "environments," focusing on the essential nature and scope of the impact of everyday life design. It reflects how changes in individual differences impact the present and future of everyday life design through environmental, behavioral, and cultural heritage recordation [[46], [47], [48], [49]].

Cluster 3 includes keywords such as "daily life," "daily-life," "diary," and "personality," mainly serving as synonyms for everyday life. It involves multidimensional research on everyday life from various fields, with a rich vocabulary concerning the fields, but with a higher frequency in the fields of medicine (especially for vulnerable groups) and psychology [[50], [51], [52]].

### 3.6 Burst terms

The term "burst terms" describes vocabulary with significant frequency changes over a short period, effectively portraying the research frontiers and trends at each stage. In the burst terms of the design history of everyday life (Table 3), "public space" exhibits the highest burst intensity (3.08), while "humanization" shows the lowest (0.96). Based on their temporal characteristics, burst terms can be categorized into three stages: the first stage (1990–2000) saw no relevant burst terms; the second stage (2000–2010) witnessed the highest burst rates for "redesign," "consumption," and "aestheticization"; the third stage (2010–2022) has been marked by the lasting impact of "historic district," "sense of place," and "aesthetic criticism," especially the enduring influence of "public space" up to the present day. Our review of the publications indicates that in China, architectural design and urban planning increasingly prioritize the perspective of everyday life, particularly in the revitalization of old urban areas. Overall, from 2006 to 2022, over this 16-year period, the research perspective on the design history of everyday life has been remarkably diverse.

**Table 3:** Burst terms in the design history of everyday life research publications 1990–2022.

Burst terms	Years	Strength	Begin	End	Burst time(1990–2022)
culture	2006	1.72	2006	2012	
lifestyle	2006	1.29	2006	2012	
industrial design	2007	1.44	2007	2010	
post-modern	2007	1.07	2007	2010	
aestheticization	2004	1.93	2008	2010	
consumption	2008	1.93	2008	2012	
design method	2009	1.43	2009	2011	
redesign	2010	2.27	2010	2012	
innovate	2010	1.63	2010	2012	
instrumental rationality	2010	0.99	2010	2013	
China	2011	1.12	2011	2013	
art of design	2012	2.18	2012	2014	
product design	2007	1.58	2012	2015	
tradition	2012	1.09	2012	2014	
create	2012	1.09	2012	2014	
architectural design	2013	1.59	2013	2015	
Kenya Hara	2013	1.37	2013	2016	
aesthetic criticism	2014	1.34	2014	2017	
feature	2014	1.14	2014	2015	
functionalism	2014	1.14	2014	2015	
art design	2008	2.97	2016	2017	
historic district	2017	2.18	2017	2020	
place spirit	2018	0.99	2018	2020	
public space	2019	3.08	2019	2022	
humanization	2019	0.96	2019	2020	

^3^ The red bold line indicates the start and end years of significant burst terms, while the bright green midline represents relatively significant start and end years. The dark green thin line indicates years with no significant burst terms.

## 4 Additions and improvements

The limitations of bibliometrics as a research method based on mathematics and statistics include the insufficient acquisition of information, which fails to effectively reveal the macroscopic regularities in the research field [53]. For instance, the leading journal "Journal of Design History" not appearing in the overall bibliometric analysis reflects the presence of omitted variable bias in bibliometrics. Therefore, the selection of JDH is essential to supplement the journal publications research.

By categorizing the keywords into three stages, the analysis reveals the changing trends in research hotspots, as shown in Table 4. The significant research areas in the history of design in the past decade have been in material culture. In the study of the design history of everyday life, there is a significant emphasis on "women," including female designers, female domestic workers, women's magazines, and female muralists.

1) The keywords "design history" and "technical history" have shown relatively stable changes during this stage, indicating JDH's consistent focus on the art and technical history encompassed within design history. Across the entirety of the journal's publications, authors from diverse geographical regions depict a comprehensive spectrum of designs and seek new ways to rewrite and create design history. The research domain has continuously expanded, research methods have become more enriched, and the research subjects have become increasingly detailed and marginalized, from the founding of the journal in 1988, with a focus on the development of national design organizations, to the more "remote" design perspectives in 2022. This progression includes analyses of influential figures, explorations of vernacular architecture, and perspectives on men's fashion and the handicrafts of disabled men [[54], [55], [56]].
2) The keywords "18th/19th century," "popular culture," and "decorative arts history" have experienced a decline in ranking from 1990 to 2022. Nevertheless, the influence of everyday life has not diminished but rather increased. In the study of design history in the 18th and 19th centuries, Sally-Anne Huxtable, for instance, delves into the complex and symbolic meanings of the color white in art and design based on "white walls, white nights, and the White Lady" [57]. More scholars have placed popular culture and decorative arts history within specific time periods of the 18th and 19th centuries, with specific focus on the exhibition held in Prague in 1895 to highlight folk culture and the shift of decorative arts history from high-end crafts and elaborate imagery to the household crafts and art at the end of the 19th century in England, including the voices of those overlooked by decorative arts history [58].
3) The keywords "household," "material culture," "women," "oral history," and "everyday life" have experienced an increase in ranking during this stage. The study of interior design history from the perspective of everyday life has become an important research direction in architectural history. For example, Lisa Godson's research on rural furniture in Ireland reveals two ways of life in Ireland and places the study of vernacular architecture in a position of significance [59]. Since 2011, the combined study of women and the design history of everyday life has become particularly prominent. Burman and Fennetaux, for example, used various sources such as publications, diaries, letters, advertisements, portraits, and criminal records to collect 390 public and private pocket collections, documenting the forgotten or neglected lives of women [60]. Scholars have also focused on Bauhaus women, opening up a new research perspective on the pioneering design school Bauhaus [61]. In material culture studies related to the design history of everyday life, seemingly ordinary items such as underwear, chests of drawers, and Easter have gradually become focal points of design history, continuously permeating the scope of everyday life.

**Table 4:** Important keywords in Journal of Design History during 1990–2022.

Important keywords	Total		1990–2000		2001–2010		2010–2022	
	Times	Rank	Times	Rank	Times	Rank	Times	Rank
Design history	32	1	4	2	15	1	13	2
Domestic	30	2	3	4	13	4	14	1
18th-19th Centuries	28	3	6	1	14	3	8	3
popular culture	25	4	4	2	15	1	6	5
material culture	19	5	3	4	8	7	8	3
crafts history	15	6	3	4	10	5	2	9
Women	13	7	2	7	5	8	6	5
gender politics	12	8	1	9	10	5	1	11
history of technology	8	9	2	7	2	9	4	7
oral history	6	10	1	9	2	9	3	8
Everyday life	3	11	–	–	1	11	2	9

^4^ For the statistical analysis of the JDH journal, it is noted that some keywords are not highly relevant to this study. The keywords in this table have been obtained through a secondary screening process.

## 5 Discussion

This study provides a comprehensive review of the research progress in the design history of everyday life, employing bibliometric analysis and knowledge graph methods. The key findings are as follows:

There is an upward trend in the number of publications on the design history of everyday life, with a predominant contribution from the United Kingdom and the United States. Journals in the field tend to be inclined towards history and architecture. Key research organizations include comprehensive universities, art schools, and select medical organizations.

The emphasis of the design history of everyday life varies across different periods. Analysis of high-frequency keywords reveals that initial research focused on practical issues of everyday life design, such as healthcare and architecture, without venturing into the realm of design history. After 2005, there was a greater emphasis on specific studies of design objects related to everyday life. Thematic trend analysis identifies topics such as retrospective exploration of everyday life, living spaces, and education as having significant potential. The knowledge graph analysis of keywords indicates that current research in the design history of everyday life is centered around aspects such as consumption, behavioral prediction, personalization, and the exploration of social modernity. Citations and analysis from the Journal of Design History suggest that the design history of everyday life has evolved from nascent awareness to specific case studies, expanding its research scope from a focus on the female perspective to various marginalized aspects.

From the current research progress, it can be observed that while there have been varying degrees of research on the design history of everyday life, it fundamentally aligns more with practical aspects of everyday life design rather than design history per se. Although there have been attempts to explore the history of everyday life from a design perspective, theoretical research in this area is still lacking in depth. Therefore, it is crucial to focus on establishing a solid theoretical framework, enhancing the influence of the design history of everyday life, delving into comprehensive research of historical materials, and seeking research methodologies for the design history of everyday life. This could include in-depth exploration of the origins and development of everyday life, as well as actively learning and drawing inspiration from other fields such as philosophy, history, anthropology, and architecture. Additionally, while thoroughly examining the development of the design history of everyday life, it is important to build upon focused and meticulous case studies.

It is noteworthy that this study reveals a close connection between the design history of everyday life and psychology. This connection is manifested in the inherent link between the human behaviors, emotions, and cultural values portrayed in the design history of everyday life, and the individual cognition, emotional experiences, and behavioral decisions studied in psychology. This relationship not only provides us with a deeper understanding of the psychological motivations behind historical design, but also enriches psychological theories with specific cultural and historical contexts.

In summary, this paper provides a comprehensive and holistic review of the research development in the design history of everyday life, utilizing bibliometric analysis and knowledge graph methods. Not only does it offer a more comprehensive and insightful reflection of design itself, but it also expands the research perspectives within the field of design history. We eagerly anticipate the design history of everyday life to showcase a diverse and outward expression, infused with a renewed vitality.

However, like any research method, bibliometric research methods have their limitations. These primarily include inconsistent data quality (publication quality, language differences, overlooking other niche research outcomes, data lag, etc.), and the fact that different authors might follow different standards, making it challenging to achieve standardization and homogenization in academic research. As a result, the research findings may not fully reflect the overall level of the field. Therefore, bibliometrics is regarded as one of the multiple tools for assessing academic research, not as the sole indicator of research quality or impact. The results of this study aim to serve as a reference for researchers interested in or working in this field. We look forward to the continuous refinement and development of bibliometric methods. By introducing more evaluation indicators and conducting in-depth data analysis, we can strive to overcome these limitations, providing a more comprehensive and diverse research perspective. In our future research, we will continue to explore and develop more specific and detailed research methods, more accurately capture the research dynamics in the field of design history of everyday Life, foresee and guide new research directions, and better serve the research needs of humanities and social sciences.

## Data availability statement

Data are available upon request or are downloadable from the Web of Science.

## Funding

This research received no external funding.

## Declaration of competing interest

The authors declare that they have no known competing financial interests or personal relationships that could have appeared to influence the work reported in this paper.
